# Latex Microsphere-Based Bicolor Immunochromatography for Qualitative Detection of Neutralizing Antibody against SARS-CoV-2

**DOI:** 10.3390/bios12020103

**Published:** 2022-02-07

**Authors:** Zhanwei Liang, Tao Peng, Xueshima Jiao, Yang Zhao, Jie Xie, You Jiang, Bo Meng, Xiang Fang, Xiaoping Yu, Xinhua Dai

**Affiliations:** 1 College of Life Sciences, China Jiliang University, Hangzhou 310018, China; s20090710033@cjlu.edu.cn (Z.L.); s20090710020@cjlu.edu.cn (X.J.); 2 Technology Innovation Center of Mass Spectrometry for State Market Regulation, Center for Advanced Measurement Science, National Institute of Metrology, Beijing 100029, China; pengtao@nim.ac.cn (T.P.); zhaoy@nim.ac.cn (Y.Z.); xiejie@nim.ac.cn (J.X.); jiangyou@nim.ac.cn (Y.J.); mengbo@nim.ac.cn (B.M.); fangxiang@nim.ac.cn (X.F.)

**Keywords:** neutralizing antibody, latex microspheres, lateral flow immunoassay, SARS-CoV-2, receptor binding domain

## Abstract

Neutralizing antibody (NAb) is a family of antibodies with special functions, which afford a degree of protection against infection and/or reduce the risk of clinically severe infection. Receptor binding domain (RBD) in the spike protein of SARS-CoV-2, a portion of the S1 subunit, can stimulate the immune system to produce NAb after infection and vaccination. The detection of NAb against SARS-CoV-2 is a simple and direct approach for evaluating a vaccine’s effectiveness. In this study, a direct, rapid, and point-of-care bicolor lateral flow immunoassay (LFIA) was developed for NAb against SARS-CoV-2 detection without sample pretreatment, and which was based on the principle of NAb-mediated blockage of the interaction between RBD and angiotensin-converting enzyme 2. In the bicolor LFIA, red and blue latex microspheres (LMs) were used to locate the test and control lines, leading to avoidance of erroneous interpretations of one-colored line results. Under the optimal conditions, NAb against SARS-CoV-2 detection carried out using the bicolor LFIA could be completed within 9 min, and the visible limit of detection was about 48 ng/mL. Thirteen serum samples were analyzed, and the results showed that the NAb levels in three positive serum samples were equal to, or higher than, 736 ng/mL. The LM-based bicolor LFIA allows one-step, rapid, convenient, inexpensive, and user-friendly determination of NAb against SARS-CoV-2 in serum.

## 1. Introduction

Severe acute respiratory syndrome coronavirus 2 (SARS-CoV-2) has spread globally over the past two years, causing pneumonia disease since 2019 (COVID-19) and resulting in significant morbidity and mortality [1]. SARS-CoV-2 particles contain four structural proteins, namely, nucleocapsid protein, envelope protein, membrane protein, and spike (S) protein. During infection, SARS-CoV-2 entry to cells depends on the S protein, which contains the receptor binding domain (RBD), mediating binding to the viral receptor of human angiotensin-converting enzyme 2 (ACE2) [2,3,4,5,6,7]. Fortunately, a variety of vaccines have been developed and used to protect humans against SARS-CoV-2. Studies on vaccinated subjects have demonstrated that the human body has the ability to rapidly induce a protective immune response and produce neutralizing antibodies (NAb) after vaccination, which affords a degree of protection against infection and/or reduces the risk of clinically severe infection [8,9,10,11,12,13,14,15,16,17]. S protein RBD of SARS-CoV-2 is the main protein that stimulates the human immune system to produce Nab [17,18,19]. 

The NAb against SARS-CoV-2 detection is a direct approach to evaluating the effectiveness of vaccines. Currently, virus neutralization testing is the gold standard for NAb detection [20,21]. However, it is highly technical, often time consuming, and high risk, because live virus- or pseudovirus-based neutralization testing is restricted to biosafety level 3 and 2 facilities. Although an enzyme-linked immunosorbent assay (ELISA) has also been developed for accurate and sensitive detection of SARS-CoV-2 antibodies, its labor- and time-consuming operation limits its widespread application on site [22,23,24]. In practice, a direct and rapid detection method without pretreatment is deserved for point-of-care testing of NAb among vaccine recipients.

A lateral flow immunoassay (LFIA), which combines chromatography technology with conventional immunoassay and nanomaterials, is considered the most attractive point-of-care testing device, because it exhibits several advantages, including simple technical requirements, rapid detection capability, portability, affordability, high detection accuracy, and high efficiency [25]. LFIAs have been widely applied in detecting pesticide [26,27] and veterinary drugs residues [28,29], toxicants [30,31,32], pathogens [32,33], various diseases biomarkers, as well as SARS-CoV-2 antigen/antibody [34,35]. Usually, the results of a LFIA are presented with the test and control lines showing the same color, resulting in it not being easy for users to discern the locations of test and control zones. If only one colored line appears, the result is interpreted as a defective test with only test line signaling, or as positive/negative result with only control line staining. Therefore, test and control lines of LFIA with different colors could avoid the misinterpretation of a result with only one colored line. Gold nanoparticles in different shapes exhibiting multicolor optical properties have been used for this purpose, a universal bicolor LFIA with blue gold nanoflowers in the test zone and red gold nanospheres in the control zone was designed by Dzantiev’s group [36]. In addition, latex microspheres (LMs) with rich and diverse colors can also be applied, especially, the abundant carboxyl groups on the surface of LMs that allow stable preparation of probes. In this study, a bicolor LFIA, based on red and blue LMs, was designed, in which the interaction between RBD and ACE2 can be blocked by the NAb without appearance of the red band. The LM-based bicolor LFIA allows one-step, rapid, convenient, inexpensive, and user-friendly determination of NAb against SARS-CoV-2 in serum.

## 2. Materials and Methods

### 2.1. Reagents and Instruments

ACE-2 recombinant protein and RBD recombinant protein were purchased from OkayBio (Nanjing, China). Polyvidone (PVP), sucrose, ProClin™ 300, and bovine serum albumin were obtained from Sigma-Aldrich (Germany). N-Hydroxysulfosuccinimide sodium salt (NHS), N-(3-Dimethylaminopropyl)-N′-ethylcarbodiimide hydrochloride (EDC), polyethylene glycol (PEG-20,000), and TWEEN-20 were purchased from Aladdin (Shanghai, China). Reference material of Nab with purity higher than 95% was provided by Suzhou novoprotein (Suzhou, China). 2-Morpholinoethanesulfonic Acid (MES) was obtained from TCI (Shanghai, China). 400 nM Carboxylated LMs (Blue/Red) were provided by Magsphere (Pasadena, CA, USA). Ultrapure water (18 MΩ cm at 25 °C) purified with a Milli-Q system from Millipore Corp. (Bedford, MA, USA) was used for solution preparation.

UniSart CN 140 nitrocellulose membranes were obtained from Sartorius (Shanghai, China). Glass fiber Pads SB-08, XYZ 3D film spraying instrument, CNC cutting machine (CTS300), and microcomputer automatic cutting machine (ZQ2402) were supplied by Kinbio Tech Co., Ltd. (Shanghai, China).

The blank serum used to optimize the parameters was purchased from Sigma. The 10 negative samples were obtained from Wuhan Jinyintan Hospital and stored at −80 °C until use. The 3 positive samples were donated by the vaccinated with informed consent.

### 2.2. Preparation of RBD-Labeled Red LMs and IgG-Conjugated Blue LMs

The RBD-labeled red LMs (RLM@RBD) and IgG-conjugated blue LMs (BLM@IgG) were prepared by the active ester method. With gentle stirring, 5 μL of blue or red LMs was added to 1 mL of MES buffer (50 mM, pH 6.0), then 10 μL of freshly prepared EDC solution (1 mg/mL) and 10 μL of NHS solution (1 mg/mL) were sequentially added into the above LMs solution. After activation for 15 min at room temperature, LMs were centrifuged at 10,000 rpm for 15 min at 4 °C, and the precipitate was dissolved in 1 mL of PBS buffer (0.01 M, pH 7.4). Recombinant RBD protein and mouse IgG were diluted to 0.04 mg/mL and 0.1 mg/mL with PB buffer (0.01 M, pH 7.7), respectively. Then, 100 μL of protein dilution was added into the LMs solution and mixed thoroughly. After incubation for another 30 min, 100 μL of 20%BSA (*w/v*) was added dropwise to block unbound sites for 15 min. Finally, the mixture was centrifuged at 10000 rpm for 15 min at 4 °C, the precipitate was resuspended with 200 μL of 0.01 M PBS buffer containing 0.5% polyvinylpyrrolidone K30, 10% sucrose, 1% BSA, and 0.05% ProClin300 and stored at 4 °C until use.

### 2.3. Preparation of LFIA Test Strips

The ACE2 recombinant protein and goat anti-mouse IgG with the appropriate concentration were sprayed on the NC membrane as a test line (T line) and control line (C line), respectively. The distance between the T and C lines was 5.0 mm, and then the prepared NC membranes were placed and dried at 37 °C for 12 h. The sample pad was immersed in the treatment solution (0.01 M of PBS buffer containing 0.25% PVP, 0.1% S9, 0.4% Tween-20 and 0.05% ProClin300) for 30 s, and dried at 37 ℃ for 10 h. Finally, the prepared sample pad, NC membrane, and absorbent pad were pasted onto the PVC bottom plate. The fabricated LFIA plate was cut into 3.0 mm-wide test strips, stored at room temperature, and kept dry.

### 2.4. Optimization of the LFIA Parameters

In order to obtain better performance of the bicolor LFIA, various analytical parameters were optimized: pH (4.0, 5.0, 6.0, 7.0) for RLM@RBD and BLM@IgG preparation, RBD concentration for labeling (0.01, 0.02, 0.04, and 0.08 mg/mL), and volume of RLM@RBD and BLM@IgG in each LFIA test strip (1.5, 2.0, 2.5, and 3.0 μL).

### 2.5. Test Procedure for Sample Testing

First, 10 μL of serum sample and 70 μL of dilution buffer (0.01 M of PBS containing 0.5% BSA and 0.01% Tween-20) were mixed with 2.5 μL of RLM@RBD and BLM@IgG, and incubated at room temperature, and then the mixture was added to the sample pad of the LFIA test strip. The results could be inspected to qualitatively detect NAb against SARS-CoV-2 after 9 min.

## 3. Results and Discussion

### 3.1. Principle and Validation of LM-Based Bicolor LFIA for NAb Detection

**Figure 1:**
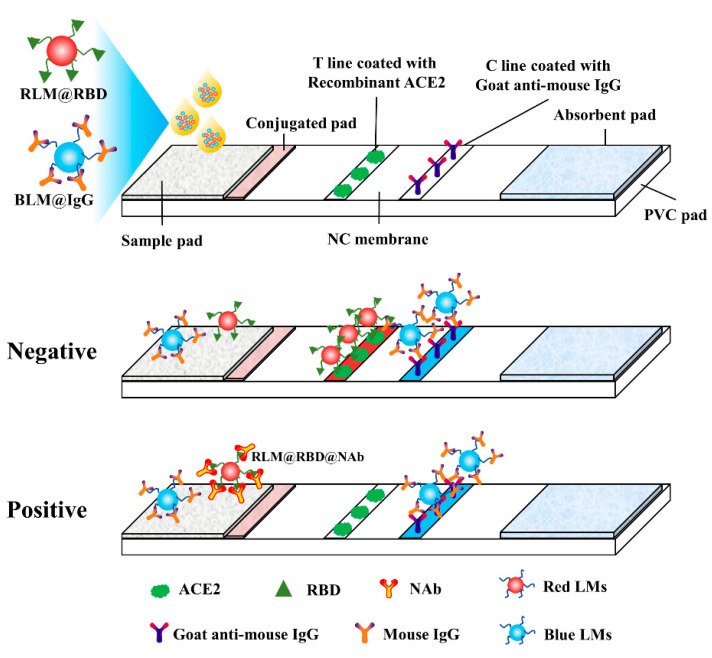
Schematic of the LFIA for anti-RBD neutralizing antibody rapid detection.

It is reported that SARS-COV-2 infection is accomplished through RBD targeting to the human ACE2 receptor, but the NAb is able to bind with the RBD and disturbs the virus–receptor engagement, contributing to protecting humans from SARS-CoV-2 infection [8,9,10,11,12,13,14,15,16,17]. The developed LM-based bicolor LFIA for NAb detection is principally based on NAb-mediated blockage of the interaction between RBD and ACE2. As shown in Figure 1, RLM@RBD and BLM@IgG are used as detection and control probes, in the absence of NAb, RLM@RBD detection probes are captured by the ACE2 coated on the T line with red band appearance, and the C line is presented as a blue band because of the conjugation between BLM@IgG control probes and goat anti-mouse IgG. On the contrary, RLM@RBD detection probes binding with ACE2 is prevented in the presence of NAb, resulting in no color appearing on the T line, but it has no effect on the blue color display of the C line. The red signal intensity on the T line is inversely correlated with the NAb levels, namely, the red signal intensity decreases with the increase of NAb levels. Moreover, the LFIA test strip is invalid in the case of no blue band appearing.

**Figure 2:**
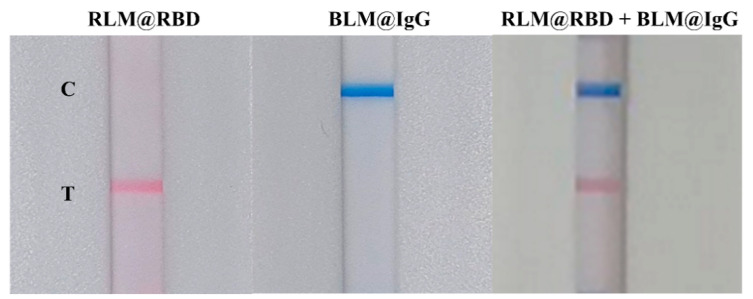
Evaluation of the interference between RLM@RBD and BLM@IgG probes.

The RLM@RBD and BLM@IgG were applied separately on the LFIA test strip to evaluate feasibility. As displayed in Figure 2, RLM@RBD and BLM@IgG were individually captured on the T and C lines with no interference with each other, and the T and C lines were presented with clear red and blue bands on the LFIA test strip when RLM@RBD and BLM@IgG were mixed. This result demonstrated that the LM-based bicolor LFIA is capable of rapid detection.

### 3.2. Optimization of the Parameters

**Figure 3:**
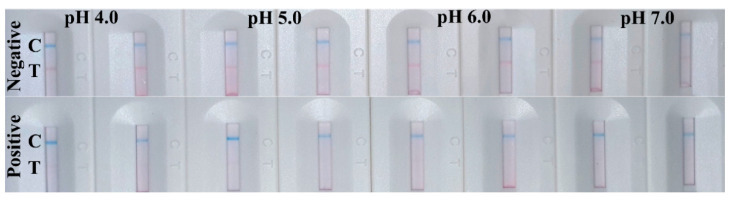
Optimization of pH for probe preparation, each measurement was tested in duplicate.

In order to obtain better performance for the bicolor LFIA, various analytical parameters were optimized. The active ester method was used to prepare RLM@RBD and BLM@IgG probes because of the abundant carboxy groups on the surface of LMs. pH is a key parameter for carboxyl group activation, which is relative to the couple efficiency between the LMs and protein. As shown in Figure 3, when the probes were prepared with pH 6.0, the test line of LFIA appeared as a clear red band with clean background on the NC membrane for negative samples. Thus, pH 6.0 was selected, which is consistent with the conclusion of a previous study [37], the carboxyl groups were better activated by EDC under a weak acid condition.

**Figure 4:**
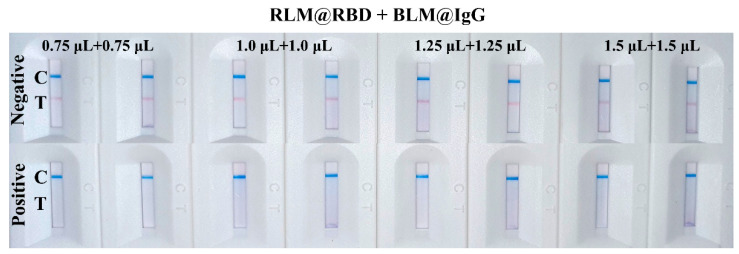
Result of the detection probe amount optimization, each measurement was tested in duplicate.

The amount of RBD protein labeled on the LMs plays a decisive role in the color development and inhibition effect of the LFIA test strip. The prepared RLM@RBD probes with different RBD protein amounts were evaluated using the blank serum and the spiked serum. The results indicated that the red signal intensity on the T line was promoted with the increase of RBD protein amount labeled on the red LMs when the blank serum was detected. However, when the RBD protein concentration was 0.04 mg/mL, the inhibition of spiked serum detection was the highest; thus, 100 μL of 0.04 mg/mL recombinant RBD protein was selected. In addition, giving consideration to the detection sensitivity and the signal intensity on the T and C lines, RLM@RBD and BLM@IgG were mixed with a ratio of 1:1 to prepare detection probes, and the volumes of the mixed detection probes were optimized. The blue signal intensity of the C line was independent of the BLM@IgG amount, but the T line showed the strongest red signal intensity when the volume of mixed detection probes was 2.5 μL, and it exhibited a good performance in detection of the spiked serum (Figure 4). Thus, 2.5 μL of mixed detection probes containing 1.25 μL of RLM@RBD and 1.25 μL of BLM@IgG were used in the subsequent experiments.

### 3.3. Performance of the Developed LM-Based Bicolor LFIA

**Figure 5:**
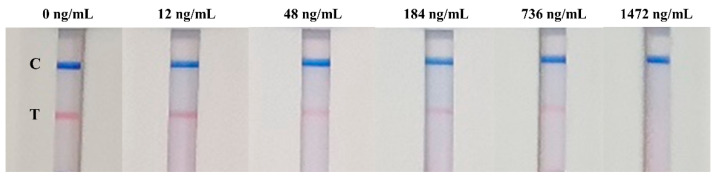
A series of spiked serum samples detected using the LM-based bicolor LFIA.

The proposed LM-based bicolor LFIA is intended to directly and rapidly detect NAb against SARS-CoV-2 in human serum. Commonly, the reaction occurring on the LFIA is instantaneous when the sample and probes migrate through the NC membrane by capillary action. The reaction time was evaluated using the negative sample, and the result obtained by naked eye showed that the detection could be completed within 9 min. In principle, the developed bicolor LFIA involved a competitive binding immunoassay, the NAb in human serum competed with the ACE2 protein on the T line for the limited binding sites on RLM@RBD. The reference material of NAb against SARS-CoV-2 was spiked into the blank human serum with the concentrations of 1472, 736, 184, 48, 12, and 0 ng/mL, and then the spiked serum samples were tested using the developed LM-based bicolor LFIA. As the result shows in Figure 5, the red signal intensity of the T line gradually weakened with increasing NAb concentration; and when the concentration was 48 ng/mL, the red signal intensity of the T line exhibited a remarkable difference compared with that of the blank serum sample. Thus, the limit of detection (LOD) for qualitative detection by the naked eye was defined as 48 ng/mL.

**Figure 6:**
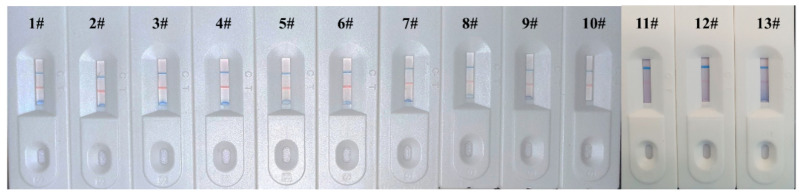
Results of the developed LM-based bicolor LFIA for 13 human serum samples point-of-care detection (1#–10# negative samples and 11#–13# positive samples).

To validate the LM-based bicolor LFIA performance in detection of real samples, 13 serum samples from non-SARS-CoV-2 infected or vaccinated healthy donors (10 negative and 3 positive) were detected. As displayed in Figure 6, the T lines of bicolor LFIA were presented with clear red bands in the case of detecting 10 negative samples, and the red bands almost disappeared when the three positive samples were tested; however, the C lines presented distinct blue bands in both cases. Comparing with Figure 5, the red signal intensity of the positive was lower than 736 ng/mL, which indicated the NAb levels in the positive serum samples may have been equal to, or even higher than, 736 ng/mL. A limitation of this work is that the number of real samples is not sufficient to validate the method, more samples will be tested to dynamically monitor the NAb level after vaccination in further work. The results demonstrated that the developed LM-based bicolor LFIA could potentially be used to directly, rapidly, and conveniently test NAb against SARS-CoV-2, which helps to predict the acquired protective immunity in COVID-19 patients or vaccines.

## 4. Discussion

NAb against SARS-CoV-2 plays a pivotal role in preventing infection and clinically severe infection, thus, NAb measurement is a good choice for the prediction of protective immunity after vaccination. Live virus- or pseudovirus-based neutralization testing assays are not implemented in routine practice. Recently, ELISA based Nab that interacts with ACE2 protein in vitro has been established [24], but the operation of ELISA is tedious, time-consuming, and depends on special equipment. Finding alternative methods without cell and virus culture operation is of interest to obtain reliable NAb information for in vitro assays, which are simple, fast, high-throughput, and commercially available. Wang et al. [38] designed a track-etched membrane microplate and portable immunosensing smartphone reader platform (TEMFIS) for simply and quantitatively detecting NAb against SARS-CoV-2, and it exhibited good performance in detecting serum/plasma samples from COVID-19 patients and vaccines. In comparison with the virus neutralization testing and ELISA, the detection by TEMFIS is simple and can be completed within 45 min. In addition, immunochromatography technique is considered an attractive point-of-care testing device, with which detection results can be obtained within 30 min.

In this proof-of-principle study, a LM-based bicolor LFIA with NAb-mediated blockage of the interaction between RBD and ACE2 was developed for detecting NAb against SARS-CoV-2 after vaccination. The bicolor LFIA benefited from the red and blue LMs used to declare the locations of the T and C lines on the LFIA test strip, leading to avoidance of the misinterpretation of only one colored line result in the traditional LFIA. Moreover, point-of-care detection of NAb against SARS-CoV-2 can be completed in 9 min without using live virus and professional equipment, which remarkably reduces the cost and improves efficiency.

## 5. Conclusions

A fully assembled LM-based bicolor LFIA was successfully developed for rapid and direct determination of NAb against SARS-CoV-2 in human serum. The red and blue LMs were used to locate the T and C lines on the LFIA; thus, the erroneous interpretation of only one colored line result, as in traditional LFIA, can be avoided. The LOD for qualitative detection by naked eye was about 48 ng/mL, and the NAb levels in three positive serum samples were detected as higher than 736 ng/mL. This point-of-care bicolor LFIA is universal and could be applied to measure other targets in multiple areas.

## Data Availability

The authors confirm that the data supporting the findings of this study are available within the article.
